# Assessing the causal association between human blood metabolites and the risk of epilepsy

**DOI:** 10.1186/s12967-022-03648-5

**Published:** 2022-09-30

**Authors:** Jiahao Cai, Xiaoyu Li, Shangbin Wu, Yang Tian, Yani Zhang, Zixin Wei, Zixiang Jin, Xiaojing Li, Xiong Chen, Wen-Xiong Chen

**Affiliations:** 1 Department of Neurology, Guangzhou Women and Children’s Medical Center, Guangzhou Medical University, Guangzhou, Guangdong China; 2 Department of Pediatrics, Nanfang Hospital, Southern Medical University, Guangzhou, Guangdong China; 3 Department of Pediatrics, Guangdong Provincial Hospital of Traditional Chinese Medicine, The Second Affiliated Hospital of Guangzhou University of Chinese Medicine, Guangzhou, Guangdong China; 4 Department of Pulmonary and Critical Care Medicine, Sun Yat-Sen Memorial Hospital, Sun Yat-Sen University, Guangzhou, Guangdong China; 5 First School of Clinical Medicine, Nanjing University of Chinese Medicine, Nanjing, Jiangsu China; 6 Department of Pediatric Urology, Guangzhou Women and Children’s Medical Center, Guangzhou Medical University, Guangzhou, Guangdong China

**Keywords:** Metabolites, Epilepsy, Mendelian randomization, Causality

## Abstract

**Background:**

Metabolic disturbance has been reported in patients with epilepsy. Still, the evidence about the causal role of metabolites in facilitating or preventing epilepsy is lacking. Systematically investigating the causality between blood metabolites and epilepsy would help provide novel targets for epilepsy screening and prevention.

**Methods:**

We conducted two-sample Mendelian randomization (MR) analysis. Data for 486 human blood metabolites came from a genome-wide association study (GWAS) comprising 7824 participants. GWAS data for epilepsy were obtained from the International League Against Epilepsy (ILAE) consortium for primary analysis and the FinnGen consortium for replication and meta-analysis. Sensitivity analyses were conducted to evaluate heterogeneity and pleiotropy.

**Results:**

482 out of 486 metabolites were included for MR analysis following rigorous genetic variants selection. After IVW and sensitivity analysis filtration, six metabolites with causal effects on epilepsy were identified from the ILAE consortium. Only four metabolites remained significant associations with epilepsy when combined with the FinnGen consortium [uridine: odds ratio (OR) = 2.34, 95% confidence interval (CI) = 1.48–3.71, *P* = 0.0003; 2-hydroxystearate: OR = 1.61, 95% CI = 1.19–2.18, *P* = 0.002; decanoylcarnitine: OR = 0.82, 95% CI = 0.72–0.94, *P* = 0.004; myo-inositol: OR = 0.77, 95% CI = 0.62–0.96, *P* = 0.02].

**Conclusion:**

The evidence that the four metabolites mentioned above are associated with epilepsy in a causal way provides a novel insight into the underlying mechanisms of epilepsy by integrating genomics with metabolism, and has an implication for epilepsy screening and prevention.

**Supplementary Information:**

The online version contains supplementary material available at 10.1186/s12967-022-03648-5.

## Introduction

Epilepsy is one of the complex and ever-present chronic diseases of neurology, pathologically characterized by sudden, abnormal electrical discharges that can lead to transient cerebral dysfunction [1]. A body of epidemiological investigations suggested that the incidence rate was 61.44 per 100,000 person-years on a global scale [2]. As estimated, epilepsy accounts for more than 0.5% of the global burden of disease, which has a substantial financial impact in terms of healthcare needs, premature death, and lost work productivity [3]. In this context, early identification and prevention of epilepsy is a high priority.

Given the possibility to take snapshots of the intricate and multivariate biochemical processes involved in illness development, investigating the relationships between metabolic abnormalities and human diseases has sparked a great deal of interest [4]. Likewise, there is a lot of research into the potential link between metabolites and epilepsy, implying that certain metabolites are involved in the development of epilepsy. Myo-inositol, for instance, has been shown in experimental experiments to have a seizure-suppressing impact, indicating a potential protective role for myo-inositol in epilepsy [5, 6, 7]. In addition, the term "metabolic epilepsy" has been proposed by the International League Against Epilepsy (ILAE) organization, which has identified numerous metabolic issues in relation to epilepsy [8]. Lin Lin Lee et al. [9] also summarized 14 metabolic disorders involved in epilepsy, like urea cycle disorders [10], glutaric aciduria [11], and so on. However, to our knowledge, there is still a paucity of comprehensive and systematic research appraising the causal effect of blood metabolites on epilepsy. Hence, owing to the inherent defects of the conventional observational studies, it is unable to conclusively delineate a metabolite spectrum contributing to the development of epilepsy based on the existing evidence.

Mendelian randomization (MR), a recently developed analytic method, has been widely applied to infer causal impacts from exposures to outcomes [12]. In the case of the absence of randomized controlled trials (RCTs) or embarking on new RCTs, the MR approach is a critical alternative strategy providing reliable evidence on the causality between exposures and disease risks [13]. Specifically, MR design leverages single nucleotide polymorphisms (SNPs) as the unconfounded instrumental variables (IVs) to proxy the phenotypes of interest. Considering the random allocation of genetic variants during fertilization, in which the process mimics an RCT, confounding (like sex and age) is less likely to bias the causal inference [14]. Besides, genotype formation happens before disease onset and is typically not affected by disease progression, thus making reverse causality less likely.

Given that the causal impacts of blood metabolites on epilepsy were poorly understood, this study utilized genome-wide association study (GWAS) statistics to systematically evaluate the potential causalities in a two-sample MR framework. To be more exploratory in identifying the prospective candidate metabolites implicated in the etiology of epilepsy, an exposure-wide design incorporating more than 400 blood metabolites was used in the present study. Findings from this work would not only help to realize the pathophysiology underlying epilepsy, but also provide reliable evidence for establishing feasible strategies for epilepsy screening and prevention in clinical practice.

## Methods and materials

### Study design

We systematically assessed the causal association between 486 human blood metabolites and the risk of epilepsy using a two-sample MR design. A convincing MR design should be in compliance with three fundamental assumptions: (1) genetic instruments are robustly associated with exposures; (2) genetic instruments are not associated with confounders; (3) genetic instruments influence the outcome only through exposures of interest [15]. Among them, the second and third assumptions are collectively known as the independence of horizontal pleiotropy, which could be tested using an array of statistical methods [16]. Genetic information for epilepsy was obtained from two independent GWAS consortia for primary analysis and replication analysis, and then a meta-analysis was performed.

**Fig. 1:**
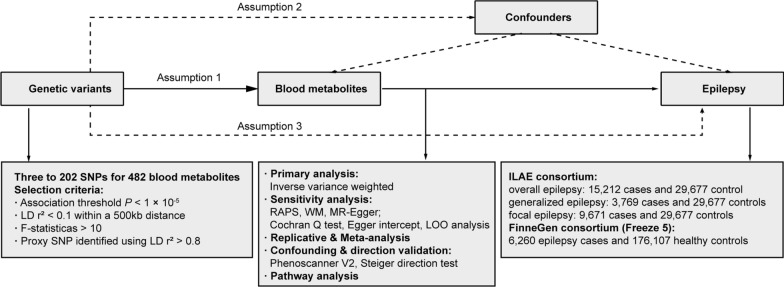
Overview of the current Mendelian randomization (MR) study. Assumption 1, genetic variants are robustly associated with exposure; Assumption 2, genetic variants are not associated with confounders; Assumption 3, genetic variants affect the outcomes only through the exposure of interest. SNPs, single nucleotide polymorphisms; LD, linkage disequilibrium; RAPS, robust adjusted profile scores; WM, weighted median; LOO, leave-one-out; ILAE, the International League Against Epilepsy.

A brief introduction to the MR approach was presented in the Supplementary materials. The study overview was presented in Fig. 1. All statistical analyses were performed using the "TwoSampleMR" package (Version 0.5.4) in the R program (Version 4.0.0), the Reviewer Manager software (Version 5.4.1), and the LD Score Regression (LDSC) software (version 1.0.1).

### GWAS data for human blood metabolites

Genetic information for each blood metabolite was obtained from the Metabolomics GWAS server (http://metabolomics.helmholtz-muenchen.de/gwas/). Specifically, the genetic variants were derived from genome-wide association scans with high-throughput metabolic profiling conducted by Shin et al. [17]. A total of 7824 European descents were included and approximately 2.1 million SNPs for 486 metabolites were tested. Among the 486 metabolites, 177 metabolites were unknown because their chemical identity has not been conclusively determined. Another 309 metabolites were chemically identified and assigned to eight broad metabolic groups, including amino acid, carbohydrate, cofactors and vitamin, energy, lipid, nucleotide, peptide, and xenobiotic metabolism, as defined in the Kyoto Encyclopedia of Genes and Genomes (KEGG) database [18].

### GWAS data for epilepsy

The GWAS statistics for epilepsy in primary analysis were obtained from the International League Against Epilepsy (ILAE) consortium, comprising 15,212 epilepsy cases and 29,677 control subjects (nearly 86% Europeans) [19]. Approximately half of the participants were female. More details about the diagnosis criteria, demography, and quality control were described in the original GWAS study. Genetic information for epileptic subtypes (generalized epilepsy: 3,769 cases and 29,677 controls; focal epilepsy: 9,671 cases and 29,677 controls) were utilized for subgroup analysis.

To validate our results by conducting replication analysis and meta-analysis, we used the epilepsy data from Freeze 5 of the FinnGen consortium (6,260 epilepsy cases and 176,107 healthy controls), which is publicly available at the website: https://gwas.mrcieu.ac.uk.

### Instruments selection

A series of steps for selecting eligible genetic variants associated with metabolites were performed. First, considering the limited number of SNPs reaching genome-wide significance, we relaxed the association threshold using *P* < 1 × 10^–5^ [pairwise linkage disequilibrium (LD) r^2^ < 0.1 within a 500 kb distance] to obtain top independent SNPs, which was in accordance with the study of Yang et al. [20]. This method was widely used in previous MR studies [, 21, 22]. Meanwhile, to avoid bias owing to the employment of weak instruments, F statistics were calculated for each SNP to measure the statistical strength as previously described [21]. SNPs with F < 10 were recognized as weak instruments and were discarded to ensure all the SNPs conferred sufficient variance for corresponding metabolites [14]. We then extracted the exposure SNPs from the outcome data and excluded those associated with the outcome (*P* < 1 × 10^–5^). For SNPs absent in the outcome, proxies were identified in high LD (r^2^ > 0.8) based on the European reference panel of the 1000 Genomes Project. For those absent and no appropriate proxies identified, we discarded them. Harmonization was then conducted to align the alleles of exposure- and outcome-SNPs, and discard palindromic SNPs with intermediate effect allele frequencies (EAF > 0.42) or SNPs with incompatible alleles (e.g. A/G vs. A/C). Finally, metabolites with more than two SNPs were kept for MR analysis [23].

### Primary analysis

The random-effect inverse variance weighted (IVW) method was conducted as the primary analysis to identify significant causal associations between metabolites and epilepsy with *P* < 0.05. IVW is the major method commonly used in MR studies, which combines all the Wald ratios for each SNP to elicit a pooled estimate [24]. Specifically, IVW assumes that all the genetic variants are valid, and hence is the most powerful method for MR estimation but also prone to pleiotropic bias. Therefore, IVW was conducted as the primary method in this study to scan preliminary associations of metabolites with epilepsy.

### Sensitivity analysis

For the identified significant estimates (IVW *P* < 0.05), sensitivity analyses were then conducted to evaluate any bias of the MR assumptions. Several other MR models, including robust adjusted profile scores (RAPS), weighted median (WM), and MR-Egger (slope term), were used as complementary methods. As the extension of IVW, RAPS allows the employment of relatively weak instruments for MR estimation [25]. WM assumes that at least half of the instruments are valid [26], while MR-Egger regression provides consistent estimates accounting for pleiotropy when all the instruments are invalid [27]. To detect the existence of heterogeneity, the Cochran Q test was carried out. Cochran-Q derived *P* < 0.05 and *I*^*2*^ > 25% was recognized as existing heterogeneity [28]. Horizontal pleiotropy was evaluated based on Egger intercepts [27]. Leave-one-out (LOO) analysis was conducted to detect high influence points driving the pooled IVW estimates.

As such, the potential eligible candidate metabolites involved in epilepsy development were determined in compliance with the following items: 1) consistent directions and magnitude among the four MR methods; 2) no heterogeneity or pleiotropy was detected; 3) no high-influence points were identified in LOO analysis.

### Replication and meta-analysis

To validate the robustness of candidate metabolites, we replicated IVW analysis using another independent epilepsy GWAS data from the FinnGen consortium mentioned above, and then conducted a meta-analysis to determine the final candidates.

### Genetic correlation and direction validation

Studies have confirmed that MR frequently generates false positives in the presence of genetic correlation between traits [, 29, 30]. Though the SNPs associated with the outcome (epilepsy) were removed throughout the instrument selection procedure, a combination of the SNPs without significant association with epilepsy could also contribute to the genetic risk of epilepsy. Thereby, to investigate whether the discovered causalities were influenced by shared genetic architecture, the genetic association between the identified metabolites and epilepsy was assessed using LDSC.

In addition, we validated whether the observed causalities were biased owing to reversed causation using the Steiger test [31]. Using the Steiger test, we determined whether the included SNPs explained more about epilepsy variability than the detected metabolites. When a combination of SNPs was found to contribute more to the genetic risk of epilepsy than metabolites (Steiger *P* > 0.05), it indicated that the direction of causal inference might be biased.

### Confounding analysis

Although an array of statistical methods were conducted in sensitivity analysis to evaluate any violation of the MR assumptions, we scanned with the Phenoscanner V2 website (http://www.phenoscanner.medschl.cam.ac.uk/) to explore whether the metabolites-associated SNPs were meanwhile associated with several common risk factors that might bias the MR estimates, including smoking [32], obesity [33], diabetes [34], and educational attainment [35]. Once the SNPs were associated with these potential confounders at the threshold of *P* < 1 × 10^–5^, IVW was replicated after dropping these SNPs to validate the robustness of the results.

### Metabolic pathway analysis

Leveraging the set of identified metabolites, metabolic pathway analysis based on the KEGG database was finally carried out using MetaboAnalyst 5.0 (https://www.metaboanalyst.ca/), a user-friendly online tool for streamlined metabolomics data analysis.

## Results

Following the instrument selection steps, 482 metabolites were kept in MR estimation (four metabolites with less than three SNPs were excluded) (Additional file 2: Table S1). The number of SNPs for each metabolite ranges from 3 to 202. F statistics for SNPs were all over 10, suggesting no weak instruments were employed (Additional file 2: Table S2). The harmonized data was presented in Table S2.

### Primary analysis

**Fig. 2:**
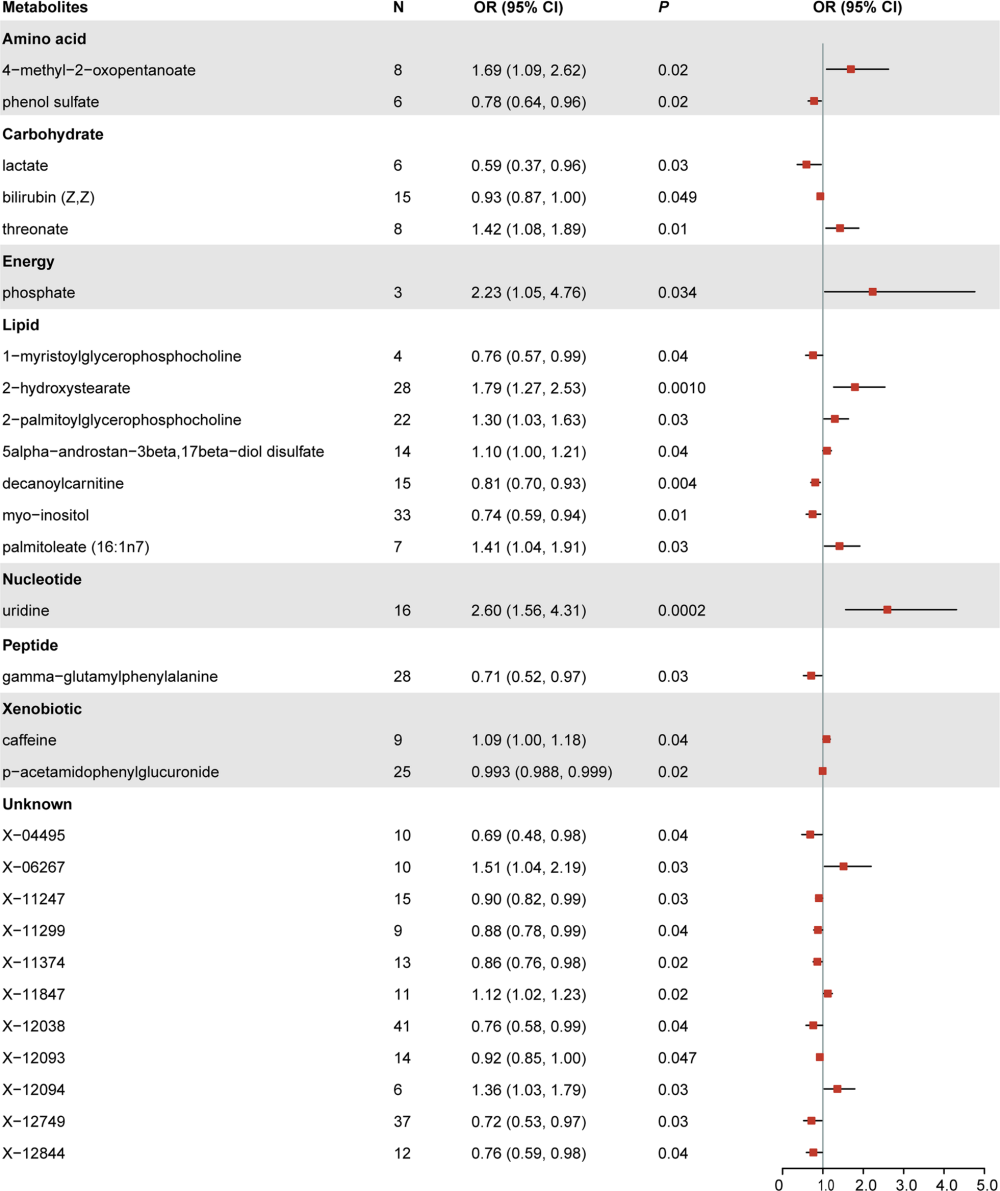
Forest plot for the causal effect of metabolites on the risk of epilepsy derived from inverse variance weighted (IVW). OR, odds ratio; CI, confidence interval.

**Fig. 3:**
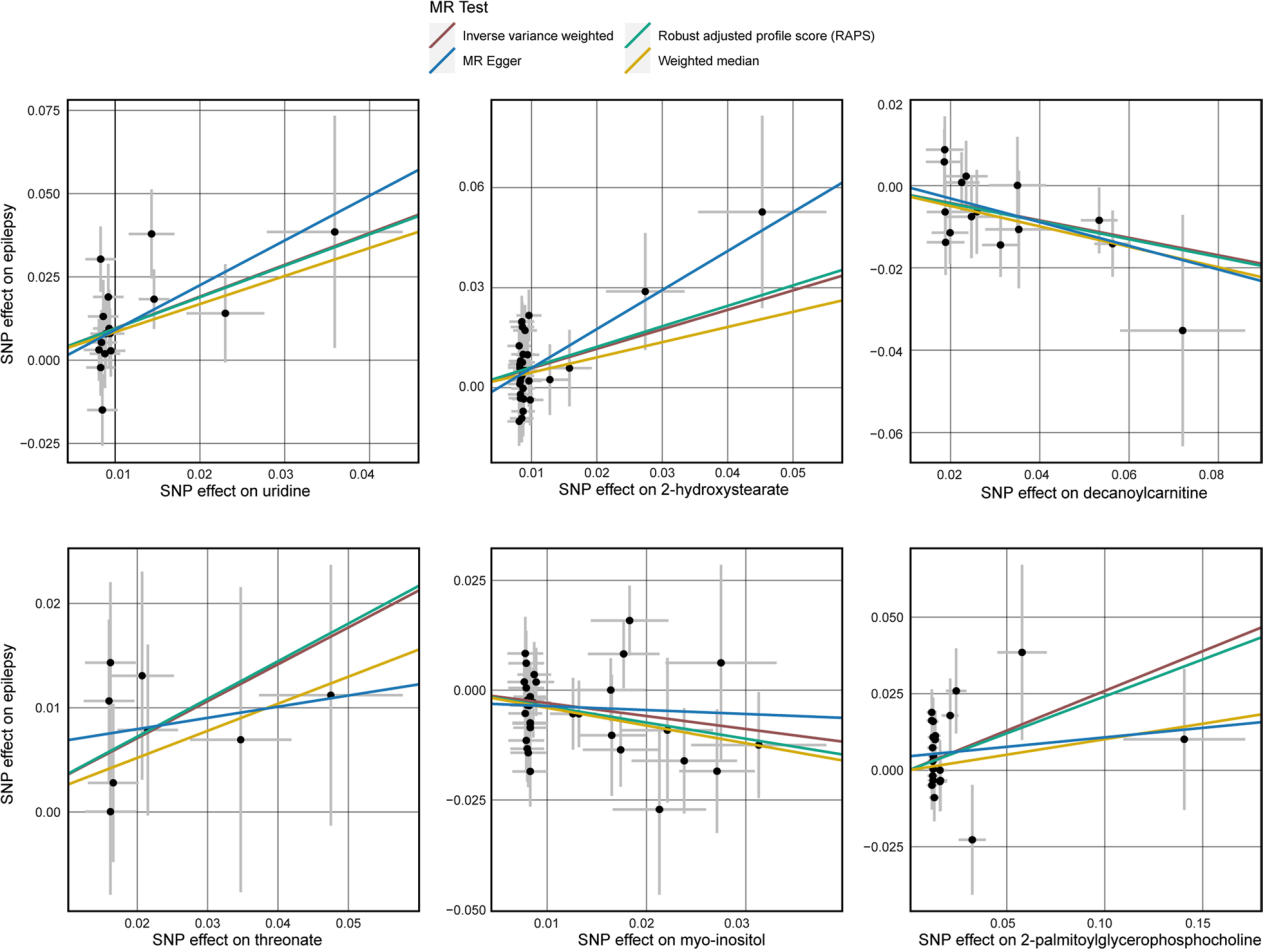
Scatterplot for the significant Mendelian randomization (MR) association (*P* < 0.05) between metabolites and epilepsy. SNP, single nucleotide polymorphism.

IVW preliminarily identified 28 metabolites significantly associated with epilepsy (Figs. 2, 3). Among them, 11 metabolites remained chemically unknown. Another 17 metabolites were chemically assigned to amino acid, carbohydrate, energy, lipid, nucleotide, peptide, and xenobiotic metabolism. By conducting sensitivity analysis, only six of them met the criteria of eligible candidate metabolites involved in the development of epilepsy, including uridine (OR = 2.60, 95% CI = 1.56–4.31, *P* = 0.0002), 2-hydroxystearate (OR = 1.79, 95% CI = 1.27–2.53, *P* = 0.001), decanoylcarnitine (OR = 0.86, 95% CI = 0.70–0.93, *P* = 0.0036), threonate (OR = 1.42, 95% CI = 1.08–1.89, *P* = 0.013), myo-inositol (OR = 0.74, 95% CI = 0.59–0.94, *P* = 0.014), and 2-palmitoylglycerophosphocholine (OR = 1.30, 95% CI = 1.03–1.63, *P* = 0.023). Briefly, MR estimates derived from RAPS, WM, and MR-Egger regression presented consistent direction and magnitude, supporting the robustness of the causality (Table 1). Cochran Q-derived *P* values and *I*^*2*^ indicated that no heterogeneity was detected. Besides, intercept terms from MR-Egger suggested a low risk of horizontal pleiotropy (Table 1). Furthermore, LOO analysis did not identify any high-influence SNPs biasing the pooled effect estimates (Additional file 1: Figure S1). These six metabolites were consequently determined as the potential candidate metabolites engaged in the pathogenesis of epilepsy for further analysis.

**Table 1:** Sensitivity analysis for the causal association between blood metabolites and epilepsy

Metabolites	N	RAPS		WM		Egger		Heterogeneity		Pleiotropy	
		OR (95% CI)	*P*	OR (95% CI)	*P*	OR (95% CI)	*P*	Q (I^2^)	*P*	Intercept	*P*
Amino acid											
4-methyl-2-oxopentanoate	8	1.71 (1.06, 2.76)	0.03	1.73 (0.96, 3.09)	0.07	1.16 (0.17, 7.98)	0.88	4.59 (0%)	0.71	0.006	0.71
Phenol sulfate	6	0.78 (0.63, 0.96)	0.02	0.85 (0.67, 1.09)	0.21	0.83 (0.45, 1.54)	0.59	6.24 (19.87%)	0.28	-0.003	0.84
Carbohydrate											
Lactate	6	0.59 (0.34, 0.99)	0.047	0.66 (0.34, 1.26)	0.21	0.33 (0.06, 1.72)	0.26	3.37 (0%)	0.64	0.01	0.51
Bilirubin (Z,Z)	15	0.94 (0.87, 1.01)	0.08	0.97 (0.89, 1.05)	0.44	1.00 (0.88, 1.14)	0.95	16.4 (14.63%)	0.29	-0.006	0.23
Threonate	8	1.44 (1.05, 1.97)	0.02	1.30 (0.89, 1.88)	0.17	1.11 (0.53, 2.36)	0.79	2.99 (0%)	0.89	0.006	0.51
Energy											
Phosphate	3	2.23 (0.94, 5.27)	0.07	2.37 (0.95, 5.88)	0.06	5.79 (0.31, 108.58)	0.45	0.49 (0%)	0.79	-0.01	0.63
Lipid											
1-myristoylglycerophosphocholine	4	0.75 (0.55, 1.02)	0.07	0.71 (0.50, 1.02)	0.06	0.58 (0.30, 1.15)	0.26	2.59 (0%)	0.46	0.009	0.5
2-hydroxystearate	28	1.85 (1.29, 2.65)	0.001	1.58 (0.98, 2.53)	0.06	3.22 (0.96, 10.80)	0.07	34.0 (20.59%)	0.17	-0.006	0.33
2-palmitoylglycerophosphocholine	22	1.27 (0.97, 1.67)	0.08	1.11 (0.80, 1.54)	0.55	1.06 (0.73, 1.54)	0.75	27.5 (23.64%)	0.15	0.005	0.21
5alpha-androstan-3beta,17beta-diol disulfate	14	1.10 (0.99, 1.21)	0.07	1.07 (0.95, 1.21)	0.28	1.10 (0.93, 1.29)	0.28	11.8 (0%)	0.54	0.0001	0.98
Decanoylcarnitine	15	0.80 (0.69, 0.94)	0.005	0.78 (0.65, 0.94)	0.01	0.75 (0.53, 1.05)	0.12	9.97 (0%)	0.76	0.003	0.64
Myo-inositol	33	0.69 (0.53, 0.91)	0.008	0.67 (0.46, 0.96)	0.03	0.91 (0.52, 1.60)	0.75	30.7 (0%)	0.53	-0.003	0.43
Palmitoleate (16:1n7)	7	1.37 (1.04, 1.80)	0.02	1.19 (0.83, 1.71)	0.34	1.20 (0.62, 2.32)	0.61	9.22 (34.92%)	0.16	0.005	0.61
Nucleotide											
Uridine	16	2.57 (1.55, 4.27)	0.0003	2.32 (1.19, 4.52)	0.01	3.83 (0.79, 18.59)	0.12	17.7 (15.25%)	0.28	-0.004	0.62
Peptide											
Gamma-glutamylphenylalanine	28	0.69 (0.50, 0.93)	0.02	0.60 (0.36, 0.98)	0.04	0.59 (0.35, 1.00)	0.06	34.2 (21.05%)	0.16	0.003	0.39
Xenobiotic											
Caffeine	9	1.09 (1.00, 1.19)	0.06	1.09 (0.98, 1.21)	0.12	1.02 (0.81, 1.29)	0.87	4.83 (0%)	0.77	0.005	0.58
p-acetamidophenylglucuronide	25	0.99 (0.99, 1.00)	0.04	1.00 (0.99, 1.00)	0.22	1.00 (0.98, 1.01)	0.52	11.1 (0%)	0.99	-0.002	0.75
Unknown											
X-04495	10	0.66 (0.44, 0.97)	0.03	0.71 (0.42, 1.18)	0.18	5.98 (0.51, 70.7)	0.19	8.92 (0%)	0.44	-0.03	0.12
X-06267	10	1.45 (1.01, 2.06)	0.04	1.41 (0.90, 2.22)	0.14	0.94 (0.40, 2.19)	0.88	12.0 (25.00%)	0.21	0.009	0.26
X-11247	15	0.90 (0.81, 1.00)	0.05	0.93 (0.81, 1.06)	0.26	0.87 (0.68, 1.10)	0.27	11.9 (0%)	0.61	0.002	0.75
X-11299	9	0.88 (0.77, 1.01)	0.07	0.93 (0.79, 1.11)	0.43	1.40 (0.62, 3.17)	0.44	8.85 (9.60%)	0.36	-0.02	0.29
X-11374	13	0.85 (0.74, 0.98)	0.03	0.85 (0.72, 1.00)	0.05	0.62 (0.35, 1.08)	0.12	6.25 (0%)	0.9	0.01	0.25
X-11847	11	1.13 (1.02, 1.25)	0.02	1.11 (0.97, 1.26)	0.13	1.23 (0.98, 1.55)	0.11	7.72 (0%)	0.66	-0.007	0.41
X-12038	41	0.76 (0.57, 1.01)	0.06	0.83 (0.57, 1.22)	0.34	1.13 (0.22, 5.80)	0.89	32.8 (0%)	0.78	-0.004	0.63
X-12093	14	0.92 (0.85, 1.00)	0.05	0.90 (0.81, 1.01)	0.08	0.94 (0.80, 1.10)	0.45	11.9 (0%)	0.54	-0.001	0.84
X-12094	6	1.36 (1.00, 1.84)	0.047	1.51 (1.07, 2.14)	0.02	1.84 (0.81, 4.16)	0.22	2.57 (0%)	0.77	-0.008	0.48
X-12749	37	0.71 (0.51, 0.97)	0.03	0.65 (0.44, 0.98)	0.04	0.41 (0.15, 1.10)	0.08	53.4 (32.58%)	0.03	0.006	0.25
X-12844	12	0.74 (0.57, 0.98)	0.03	0.72 (0.51, 1.01)	0.06	0.42 (0.18, 0.98)	0.07	9.97 (0%)	0.53	0.01	0.18

### Replication and meta-analysis

**Fig. 4:**
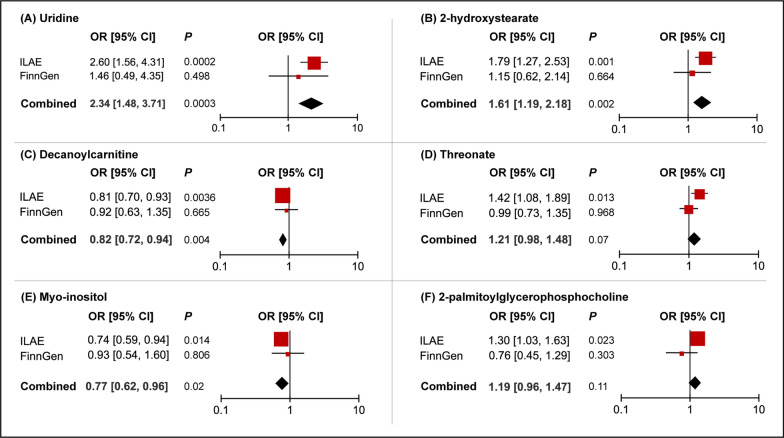
Meta-analysis of the causal associations between metabolites and epilepsy. ILAE, the International League Against Epilepsy; OR, odds ratio; CI, confidence interval.

To further verify our results, replication analysis was conducted using epilepsy GWAS data from the FinnGen consortium. As expected, similar trends were observed using FinnGen epilepsy GWAS data in certain metabolites (Fig. 4). Combined analysis of ILAE and FinnGen datasets further identified that genetic liability for higher levels of uridine (OR = 2.34, 95% CI = 1.48 – 3.71, *P* = 0.0003) and 2-hydroxystearate (OR = 1.61, 95% CI = 1.19 – 2.18, *P* = 0.002) predicted a higher risk of epilepsy, while genetic predisposition to higher levels of decanoylcarnitine (OR = 0.82, 95% CI = 0.72 – 0.94, *P* = 0.004) and myo-inositol (OR = 0.77, 95% CI = 0.62 – 0.96, *P* = 0.02) predicted a lower risk of epilepsy. Null estimates of the meta-analysis were observed in threonate and 2-palmitoylglycerophosphocholine, which yielded discordant directions using the FinnGen epilepsy database.

### Genetic correlation and direction validation

Using LDSC, we found little evidence of genetic correlation between epilepsy and 2-hydroxystearate (r_g_ = -0.001, se = 0.0334, *P* = 0.97), decanoylcarnitine (r_g_ = -0.25, se = 0.21, *P* = 0.24), and myo-inositol (r_g_ = -0.09, se = 0.13, *P* = 0.51), suggesting that the MR estimates were not confounded by the shared genetic component. For uridine, LDSC failed to detect its genetic correlation with epilepsy due to statistical characteristics of the full GWAS data (mean Chi-square = 0.97, which is inadequate for genetic correlation calculation). We further conducted a Steiger test to validate the effect direction from metabolites to epilepsy. The Steiger *P* values suggested that the identified causalities were not biased by reverse causation (Table 2).

**Table 2:** Steiger direction test from blood metabolites to epilepsy

Exposure	Uridine	2-hydroxystearate	Decanoylcarnitine	Myo-inositol
Direction	TRUE	TRUE	TRUE	TRUE
Steiger *P*	1.66 × 10^–66^	6.53 × 10^–101^	1.64 × 10^–119^	3.82 × 10^–137^

### Confounding analysis

Although sensitivity analysis found no evidence of bias invalidating the MR estimates, we further manually investigated the second traits (smoking, obesity, diabetes, and educational attainment) of the metabolite-associated SNPs. Looking over the Phenoscanner, we found that SNPs associated with uridine and myo-inositol were not associated with any of the confounders. For 2-hydroxystearate, two SNPs (rs3822742 and rs6564154) were associated with obesity-related phenotypes. After removing these two SNPs, the causality remained significant (IVW OR = 1.75, 95% CI = 1.25 – 2.44, *P* = 0.001). Similar for decanoylcarnitine, we also identified two SNPs (rs10036208 and rs11722868) were associated with obesity-related traits, and the estimates remained after discarding these two SNPs (IVW OR = 0.84, 95% CI = 0.72 – 0.98, *P* = 0.03).

### Metabolic pathway analysis

We identified five potential metabolic pathways involved in the pathogenesis of epilepsy incidence (Table 3). Myo-inositol was involved in the metabolic pathways of ascorbate and aldarate metabolism, galactose metabolism, phosphatidylinositol signaling system, and inositol phosphate metabolism (all *P* < 0.05). There was also another plausible pathway consisting of uridine, namely the pyrimidine metabolism pathway, participating in the development of epilepsy (*P* = 0.0497).

**Table 3:** Significant metabolic pathways involved in the pathogenesis of epilepsy

Pathway name	Involved metabolites	*P*
Ascorbate and aldarate metabolism	Myo-inositol	0.010
Galactose metabolism	Myo-inositol	0.035
Phosphatidylinositol signaling system	Myo-inositol	0.036
Inositol phosphate metabolism	Myo-inositol	0.038
Pyrimidine metabolism	Uridine	0.0497

### Subgroup analysis

As that GWAS data for epileptic subtypes (focal epilepsy and generalized epilepsy) were available in the ILAE consortium, we conducted subgroup analysis, expecting to preliminarily catch the first glimpses of the potential metabolites involved in the specific subtypes of epilepsy. Combined primary analysis with sensitivity analysis, we identified several candidate metabolites involved in the development of focal and generalized epilepsy, detailed in Additional file 2: Tables S3 and Additional file 2: Tables S4.

## Discussion

The current study suggested that genetic liability for higher levels of blood uridine and 2-hydroxystearate were causally associated with an increased risk of epilepsy, whereas genetic predisposition towards higher levels of decanoylcarnitine and myo-inositol played a protective role in epilepsy development. To the best of our knowledge, this is the first MR study to systematically appraise the causal role of human blood metabolites in the issue of epilepsy.

The high prevalence and recurrence of epilepsy have contributed to a heavy burden on human society, thus making disease screening and prevention extremely critical. Though some risk factors for epilepsy have been proposed, like brain infection or injury [36], the etiology of epilepsy remains unclear in nearly half of the cases [37]. Previous studies have reported several circulating biomarkers in preclinical epilepsy models [, 38, 39]. For instance, Wang et al. reported that blood matrix metalloproteinase-3 was reduced in patients with epilepsy compared with healthy controls [40]. Although that existing literature has strongly connoted the involvement of metabolic disturbance in epilepsy, current evidence is unable to conclusively determine a causal role of circulating metabolites in epilepsy development. Inspired by the metabolites GWAS analysis conducted by Shin et al. [17], we designed this exposure-wide MR study to systematically evaluate the causality between blood metabolites and epilepsy, expecting to decipher the metabolic coding underlying the epilepsy pathogenesis and provide more novel targets for epilepsy identification and prevention.

Our study suggested that genetic liability for an increased level of blood uridine and 2-hydroxystearate played a detrimental effect on epilepsy development. Few studies focused on the role of circulating uridine in the issue of epilepsy. Some observational studies reported that uridine might play a protective role in epilepsy [41], whereas some studies found no effects of uridine on epilepsy incidence [42]. These equivocal results were limited by methodological defects, like residual confounding. By leveraging MR, which is free from reverse causality and residual confounding, we found that higher levels of blood uridine predicted an increased risk of epilepsy. Previously, Slézia et al. reported an increased level of extracellular uridine in a rat model of aminopyridine-induced epilepsy, suggesting that uridine might participate in epilepsy-related neuronal activity changes [42]. However, due to the gaps in the knowledge on the biofunction of uridine, future studies are warranted to investigate the underlying mechanisms. For 2-hydroxystearate, very limited investigations have reported its association with epilepsy. A previous study reported that 2-hydroxystearate was overexpressed in diabetes patients and was positively associated with glucose levels [43]. Given that diabetes might induce epilepsy, there might be the possibility that 2-hydroxystearate causes epilepsy through diabetes, which warrants further exploration in specific experimental conditions.

Two metabolites, decanoylcarnitine, and myo-inositol, were suggested to have protective effects on epilepsy. Similarly, the literature about the role of decanoylcarnitine in epilepsy is extremely limited. Previously analysis reported the gluconeogenesis-inhibition effect of decanoylcarnitine, suggesting decanoylcarnitine might prevent epilepsy by regulating glucose metabolism [44]. However, more details for the underlying mechanisms should be explored in further study. The protective impact of myo-inositol on epilepsy identified by this MR study was supported by a great deal of literature. Using electrophysiological method, Gamkrelidze et al. found that myo-inositol has a significant local seizure-suppressant effect [5]. In a recent study, several favorable effects of myo-inositol were observed in rat models, including decreasing the frequency and duration of electrographic spontaneous recurrent seizures in the hippocampus, ameliorating epileptogenesis-related spatial learning and memory deficit, and alleviating cell loss in the hippocampus [7]. Phosphoinositide signaling pathway and myo-inositol action on gamma amino butyric acid-A receptors were the possible mechanisms of this protective effect [, 45, 46].

Taken together, the findings of our study are partially in line with those of previous studies. Based on the existing literature, the preventive role of myo-inositol in epilepsy observed in experimental studies has been well-documented. By leveraging GWAS data, our MR analysis also supported that myo-inositol exerted a protective effect on epilepsy incidence. For uridine, previous analyses yielded discrepant results, which could be attributable to the methodological flaws of the traditional observational design. Our study showed that uridine had a negative impact on epilepsy risk using the MR design, which is largely free from reverse causality and residual confounding. Furthermore, in our investigation, 2-hydroxystearate and decanoylcarnitine were discovered to be possible metabolites involved in the etiology of epilepsy. Previously, research about the role of these two metabolites in the risk of epilepsy was extremely limited. Future functional analyses were warranted to further confirm their biological effects on the development of epilepsy.

In the present study, two blood metabolites, including threonate and 2-palmitoylglycerophosphocholine, were identified as the risk factors for epilepsy using GWAS data from the ILAE consortium. However, replication analysis using epilepsy GWAS data from the FinnGen consortium yielded discrepant estimates, which might be attributed to the small proportion of epilepsy cases in the FinnGen study (~ 3.32%). Owing to the discordant results derived from two independent datasets, conclusive interpretation for the role of threonate and 2-palmitoylglycerophosphocholine in the genesis of epilepsy could not be established.

The current study has several strengths. First, the major strength worth noting in this MR study is the wide range of blood metabolites we covered. Briefly, totally 482 metabolites were included for MR analysis, which is the most comprehensive and systematic study to investigate the metabolic profiles contributing to epilepsy to date. Second, using MR design, our study is largely free from reverse causation and residual confounding. Specifically, an array of methods was implemented to verify any violation of the MR assumptions to ensure the reliability of the MR estimates. Concordant directions and similar magnitude across various MR models confirmed the robustness of the MR estimates. No evidence of horizontal pleiotropy was detected using complementary statistical methods. Third, replication and meta-analyses were applied to further support the causal effects of certain metabolites on epilepsy. Even though estimates derived from the FinnGen consortium in replication analysis were not statistically significant, the consistent directions of the effect estimates were reassuring as they appeared not to occur by chance alone. Further meta-analysis revealed several metabolites remained a significant impact on epilepsy.

Several limitations should be noted in our study. First, owing to the limited number of SNPs reaching genome-wide significance, we relaxed the *P* threshold, which is a common method widely used. The F statistic for each of the SNPs was over 10, suggesting no weak instruments were included. Besides, the Steiger test indicated a valid causal direction from the exposure to the outcome. As known, the rationale for the Steiger test is to compare the proportion of variance explained by the exposure-SNPs with that of the outcome-SNPs. Hence, the true direction derived from the Steiger test also supported the validity of the SNPs with relaxed P values. Second, the majority of the participants of this study are European. While this could largely avoid population heterogeneity, the MR results should be further validated in other populations to verify the generality in future studies when more GWAS data from other populations were publicly available. Third, despite the investigation of more than 400 blood metabolites, MR estimations were not adjusted for multiple testing in the present study. Instead, we conducted a replication analysis to verify the robustness of the MR estimates using two independent datasets (ILAE and FinnGen), greatly enhancing the credibility of our results. We argue that a conservative threshold of multiple testing might obscure the associations that were potentially noteworthy when studied alone. As such, potential candidate metabolites associated with epilepsy at *P* < 0.05 were included for further replication and meta-analyses in the present study. Finally, although that the MR approach performs excellently in causal inference, we caution that findings from this MR study should be further validated in well-powered randomized controlled trials to demonstrate the existence of causality.

In conclusion, this MR study suggests that blood metabolites might influence the risk of epilepsy in a causal way, initially providing evidence about the impact of circulating metabolic disturbance on epilepsy risk. Specifically, blood uridine, 2-hydroxystearate, decanoylcarnitine, and myo-inositol might be useful circulating metabolic biomarkers for epilepsy screening and prevention in clinical practice. These four metabolites can also serve as candidate molecules for future mechanism exploration.

## Supplementary Information

**Additional file 1: Figure S1. **Forest plots for the Mendelian randomization (MR) leave-one-out analysis of the significant inverse variance weighted (IVW) estimates. Within each panel, the black points represent the causal estimate of the association between a specific metabolite and epilepsy after discarding each SNP in turn. Red points represent the pooled IVW estimates. Horizontal lines denote 95% confidence intervals.

**Additional file 2: Table S1.** List of the identification (ID) for each of the 486 blood metabolites. **Table S2.** Harmonization data_482 blood metabolites and overall documented epilepsy from the ILAE consortium. **Table S3.** Results for the Mendelian randomization estimates and sensitivity analysis in epilepsy-subgroup (focal and generalized epilepsy) analysis. **Table S4.** Leave one out analysis for the causal association between blood metabolites and epilepsy subtypes.

## Data Availability

All data generated or during this study are included in this published article and the supplementary materials. GWAS summary statistics for human blood metabolites were publicly available at https://mrcieu.github.io/TwoSampleMR/. GWAS summary statistics for epilepsy from the ILAE consortium and FinnGen consortium were publicly available at https://gwas.mrcieu.ac.uk/.

## Abbreviations

ILAE: International League Against Epilepsy
MR: Mendelian randomization
RCTs: Randomized controlled trials
SNPs: Single nucleotide polymorphisms
IVs: Instrumental variables
GWAS: Genome-wide association study
IVW: Inverse variance weighted
RAPS: Robust adjusted profile scores
WM: Weighted median
LOO: Leave-one-out
