# Supplementary material for: Assessing the causal association between human blood metabolites and the risk of epilepsy
Source: J Transl Med. 2022 Sep 30;20:437. doi: 10.1186/s12967-022-03648-5 (PMC9524049; doi:10.1186/s12967-022-03648-5)

**Supplementary Materials**

**Assessing the Causal Association between Human Blood Metabolites and the Risk of Epilepsy**

Corresponding to Wen-Xiong Chen, Department of Neurology, Guangzhou Women and Children's Medical Center, Guangzhou Medical University, Guangdong, China. Email: gzchcwx@126.com.

**Contents**

**Supplementary Introduction to the Mendelian Randomization Approach**

**Figure S1.** Forest plots for the Mendelian randomization (MR) leave-one-out analysis of the significant inverse variance weighted (IVW) estimates.

**Supplementary Introduction to the Mendelian Randomization Approach**

Mendelian randomization (MR), a novel statistical approach for causal inference, has been widely used to investigate the risk factors for human diseases or other health-related phenotypes [1]. As known, a randomized controlled trial (RCT) is the gold standard for causality determination [2]. However, launching an RCT is typically challenging for a variety of reasons, including large time and labor expenses, ethical constraints, and so on [3]. In the absence of RCT, the MR technique is an alternative method to perform causal inference. According to the hierarchy of evidence proposed by Zuccolo and Holmes, an adequately-conducted MR study provides causal evidence with greater validity than traditional observational studies [4].

In an MR framework, single nucleotide polymorphisms (SNPs) derived from genome-wide association studies (GWAS) are used as instrumental variables (IVs) to proxy exposures of interest [5]. The random assortment of genetic variants at meiosis makes the MR design a natural analog of RCT, thus making confounding less likely [6]. Besides, reverse causality is also less plausible owing to the unidirectional information flow from DNA sequence to phenotypes (genotype formation prior to disease onset).

A well-designed MR study should meet the following fundamental assumptions [7]: i) relevance assumption: genetic variants should be significantly associated with exposures; ii) exclusiveness assumption: genetic variants are not associated with potential confounders; iii) independence assumption: genetic variants affect the outcomes only through exposures. The first assumption can be satisfied by setting the *P* threshold when retrieving the SNPs from the exposure dataset. The second and third assumptions are collectively regarded as independence from horizontal pleiotropy, which can be validated using an array of statistical methods developed recently. Horizontal pleiotropy, also known as biological pleiotropy, is a major concern in the MR approach. The existence of horizontal pleiotropy could introduce bias to the MR estimates, leading to spurious correlations. To date, complementary sensitivity analyses based on various assumptions have been established to evaluate any violation of the MR assumptions, especially for the second and third assumptions. It has been recommended to utilize various MR models, like the inverse variance weighted (IVW), weighted median, and MR-Egger regression, to complementarily detect the causal effects. Typically, the consistent direction and comparable magnitude across different MR methods enhance the evidence of causality [8]. In addition, Sensitivity analysis, including the Cochran Q test [9], MR-Egger intercept analysis [10], and leave-one-out analysis, are commonly used to detect any latent bias introduced into an MR study. With the development of the MR approach, more and more statistical methods are developed to detect the validity of the MR results, like the Steiger test [11].

In conclusion, MR is an alternative approach for causality determination in the lack of RCT. The natural advantage of the MR approach makes it less susceptible to reverse causality and residual confounding. The use of large-scale GWAS data also makes it more statistically powerful. Despite the excellent performance of MR research in causality inference, interpretation of the MR results should be cautious. We argue that results from MR research should be further validated by RCTs to ensure the existence of causality.

**References**

[1] Emdin CA, Khera AV, Kathiresan S. Mendelian Randomization. Jama. 2017;318(19):1925-6.

[2] Hariton E, Locascio JJ. Randomised controlled trials - the gold standard for effectiveness research: Study design: randomised controlled trials. Bjog. 2018;125(13):1716.

[3] Deaton A, Cartwright N. Understanding and misunderstanding randomized controlled trials. Soc Sci Med. 2018;210:2-21.

[4] Zuccolo L, Holmes MV. Commentary: Mendelian randomization-inspired causal inference in the absence of genetic data. Int J Epidemiol. 2017;46(3):962-5.

[5] Burgess S, Butterworth A, Thompson SG. Mendelian randomization analysis with multiple genetic variants using summarized data. Genet Epidemiol. 2013;37(7):658-65.

[6] Latvala A, Ollikainen M. Mendelian randomization in (epi)genetic epidemiology: an effective tool to be handled with care. Genome Biol. 2016;17(1):156.

[7] Davies NM, Holmes MV, Davey Smith G. Reading Mendelian randomisation studies: a guide, glossary, and checklist for clinicians. Bmj. 2018;362:k601.

[8] Bowden J, Davey Smith G, Haycock PC, Burgess S. Consistent Estimation in Mendelian Randomization with Some Invalid Instruments Using a Weighted Median Estimator. Genet Epidemiol. 2016;40(4):304-14.

[9] Greco MF, Minelli C, Sheehan NA, Thompson JR. Detecting pleiotropy in Mendelian randomisation studies with summary data and a continuous outcome. Stat Med. 2015;34(21):2926-40.

[10] Bowden J, Davey Smith G, Burgess S. Mendelian randomization with invalid instruments: effect estimation and bias detection through Egger regression. Int J Epidemiol. 2015;44(2):512-25.

[11] Hemani G, Tilling K, Davey Smith G. Orienting the causal relationship between imprecisely measured traits using GWAS summary data. PLoS Genet. 2017;13(11):e1007081.

**Figure S1.** Forest plots for the Mendelian randomization (MR) leave-one-out analysis of the significant inverse variance weighted (IVW) estimates.

Within each panel, the black points represent the causal estimate of the association between a specific metabolite and epilepsy after discarding each SNP in turn. Red points represent the pooled IVW estimates. Horizontal lines denote 95% confidence intervals.


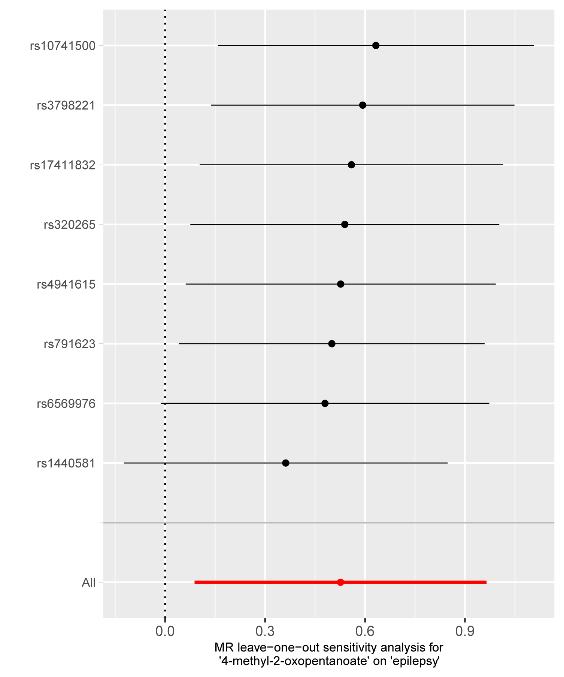

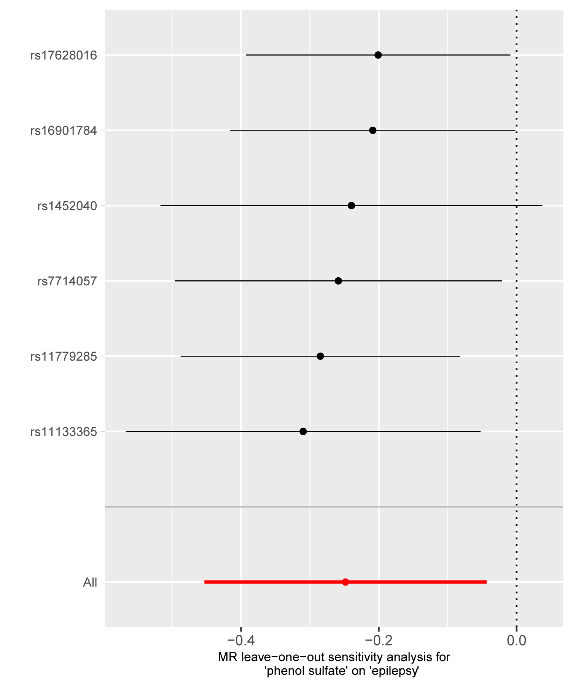


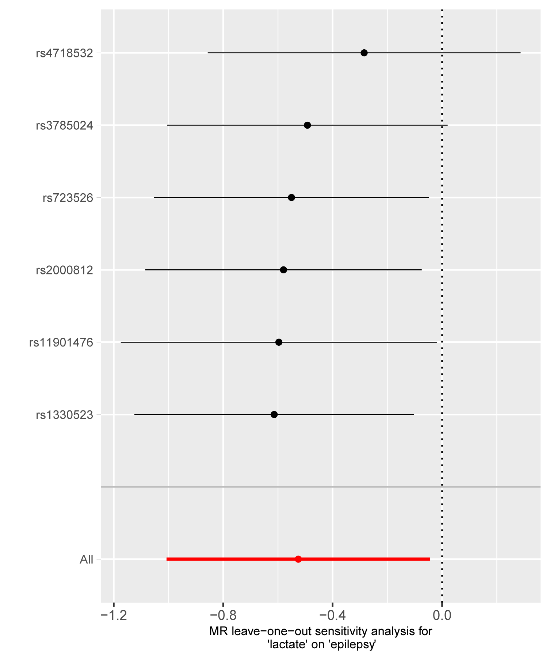

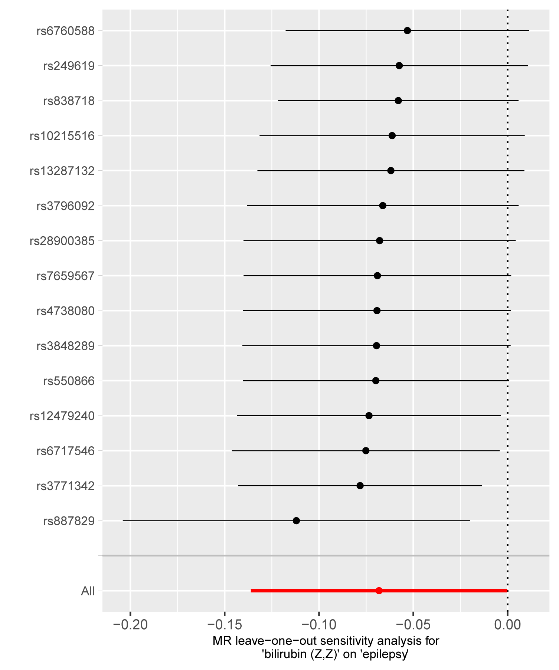

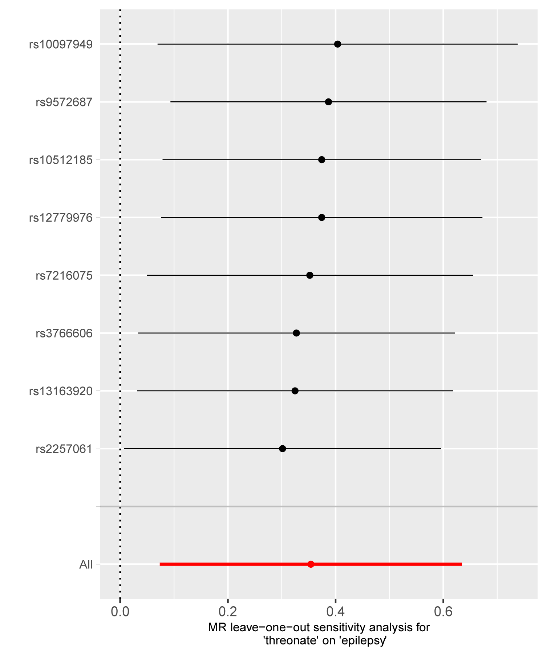

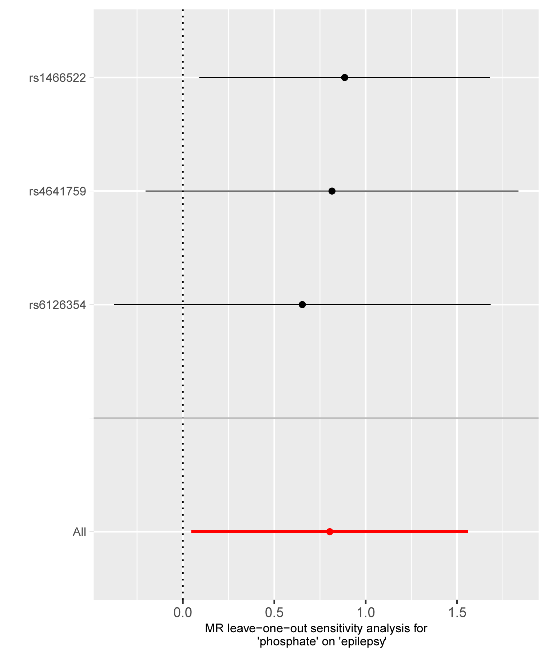

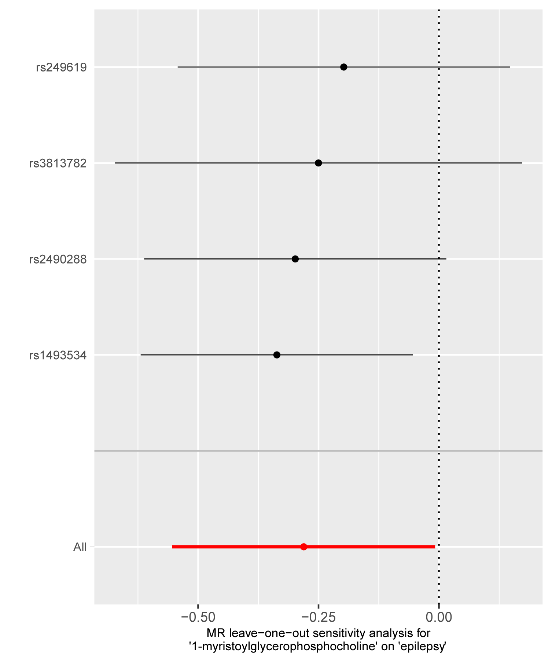

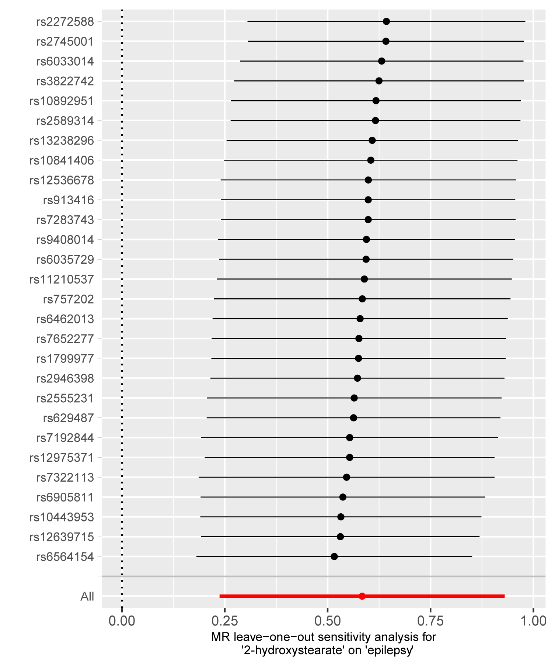

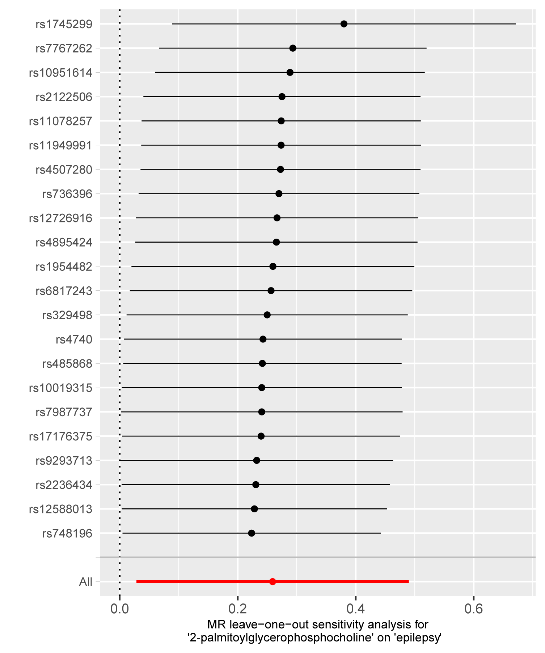

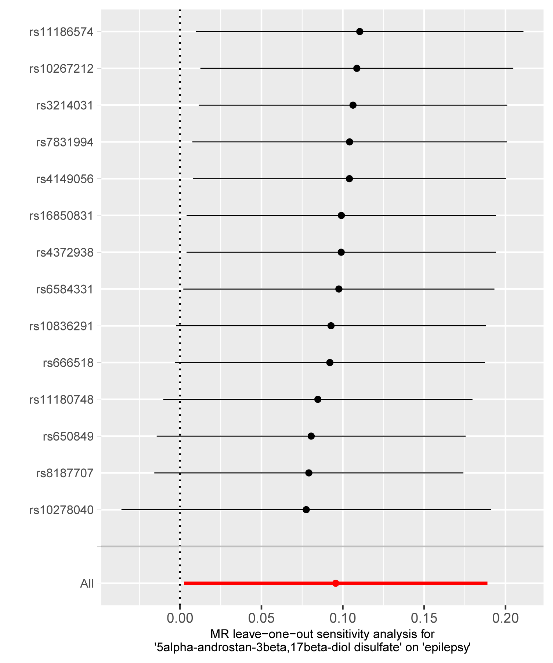

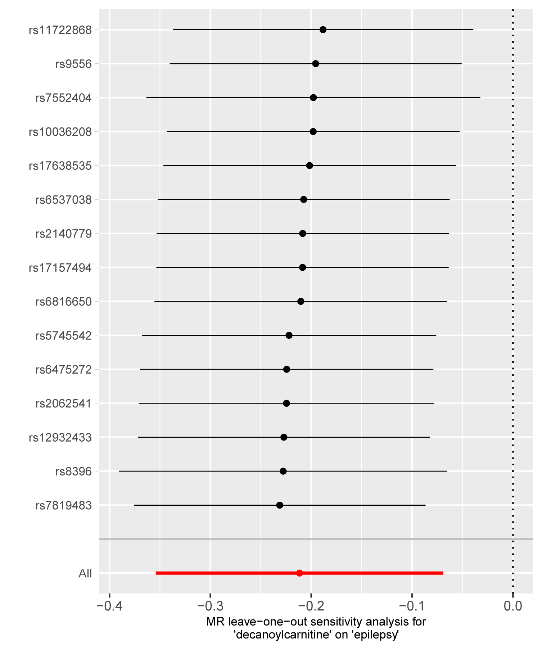

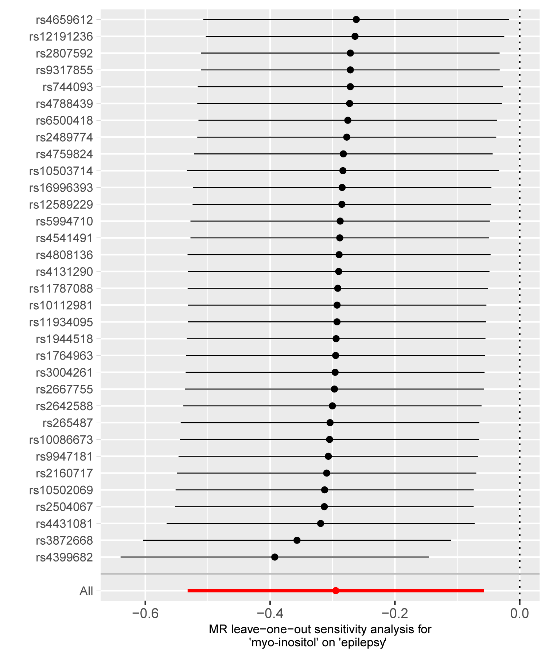

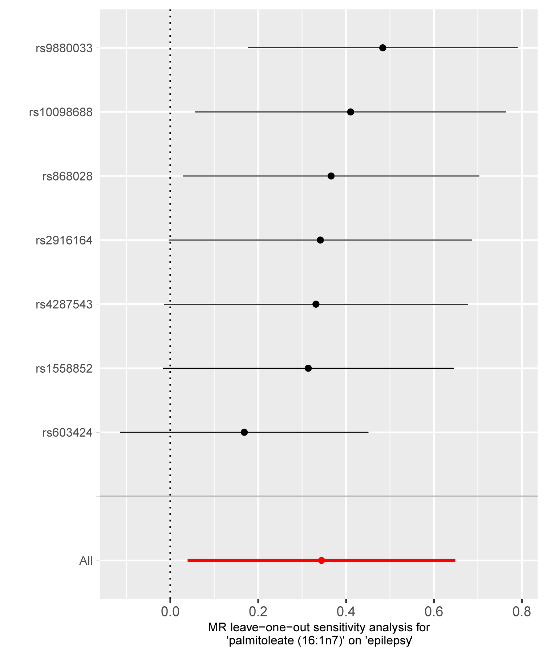

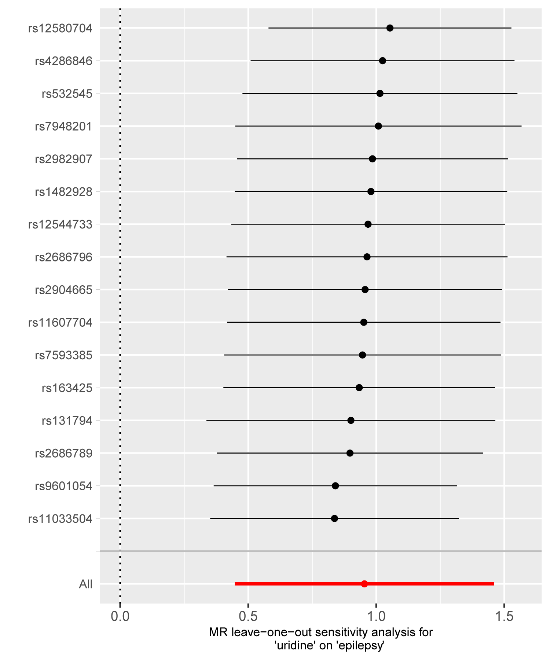

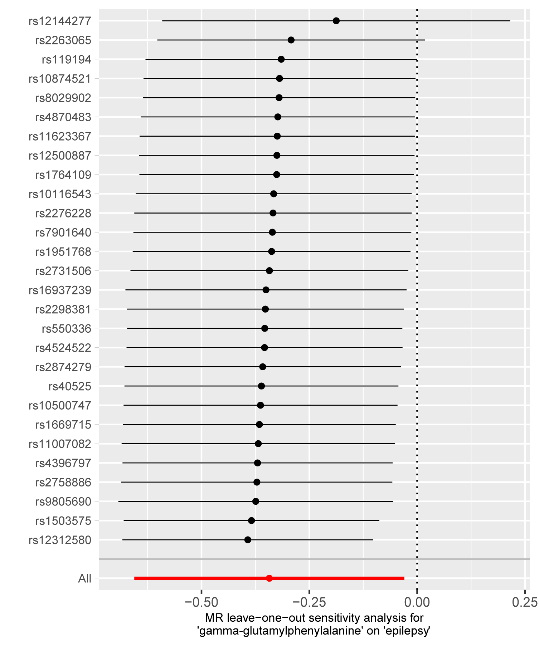

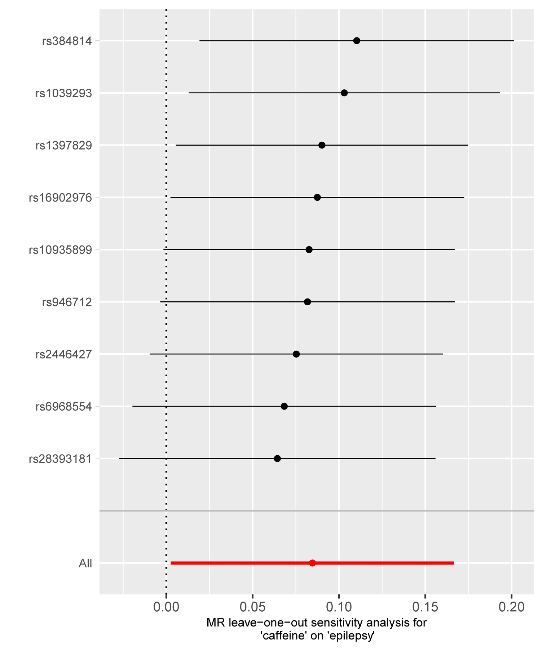

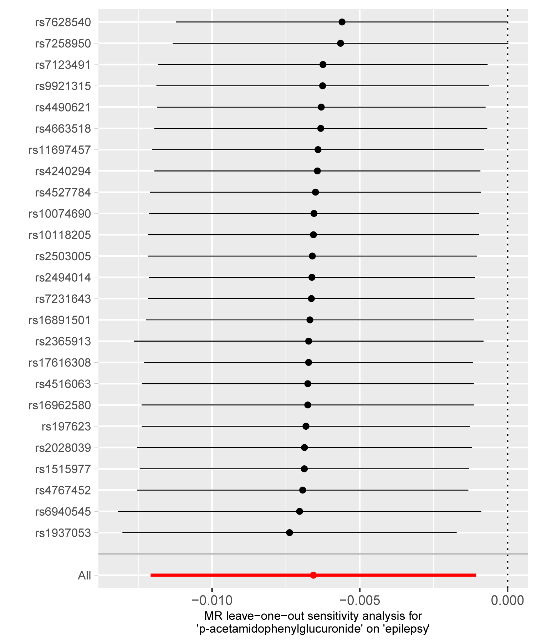

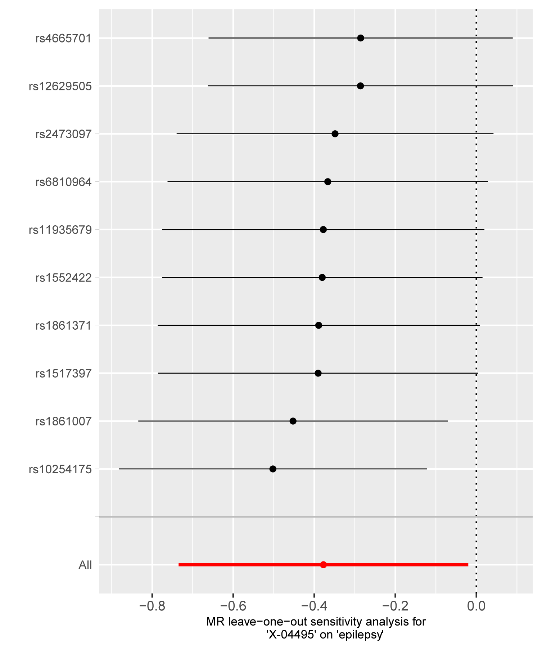

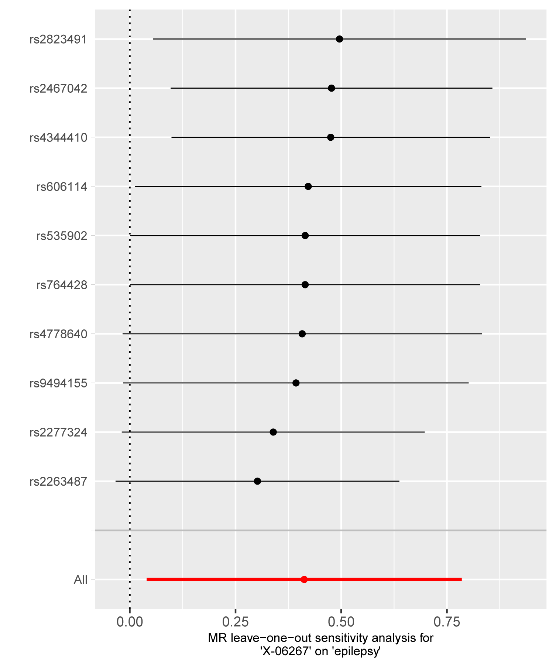

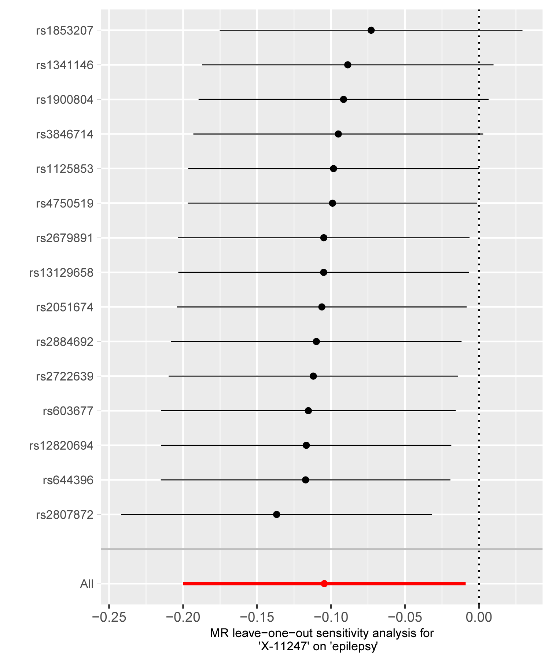

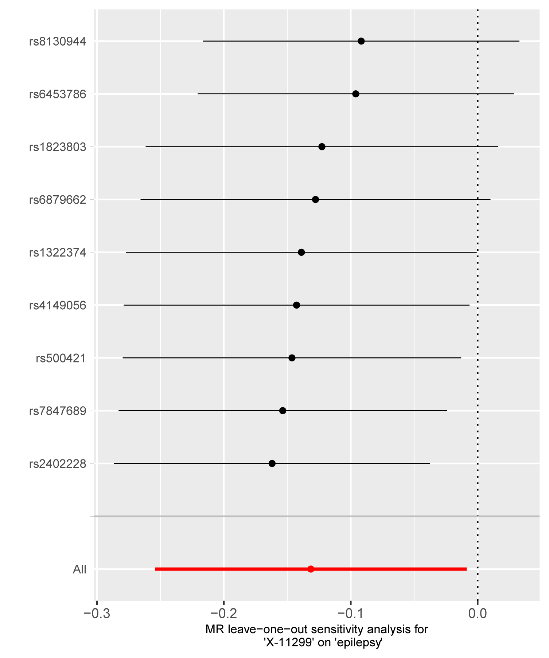

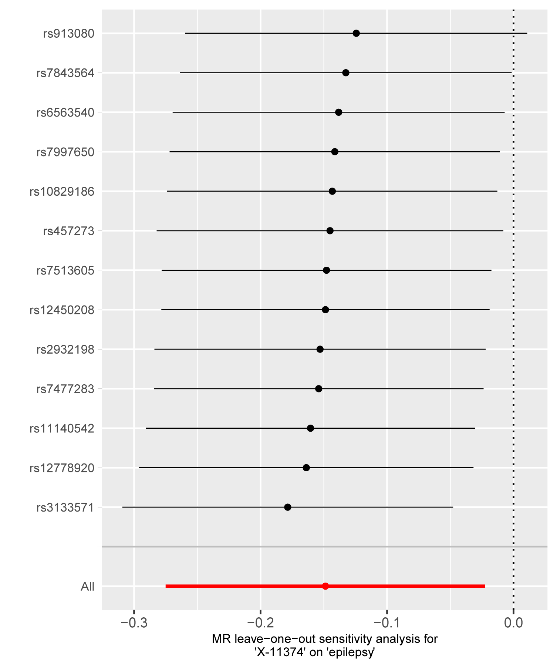

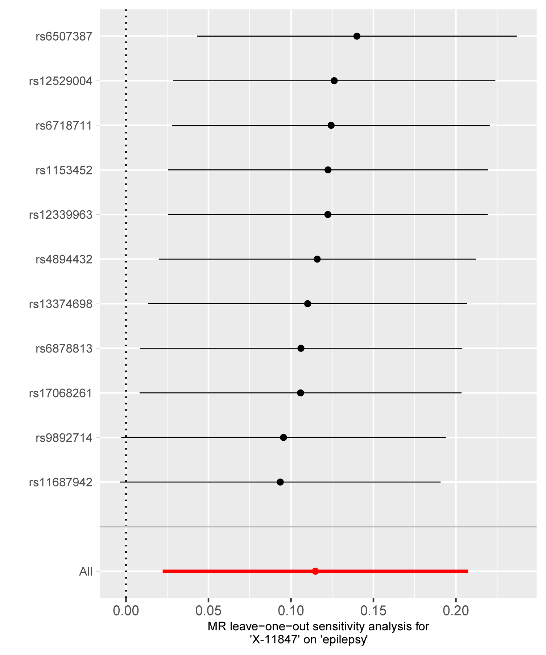

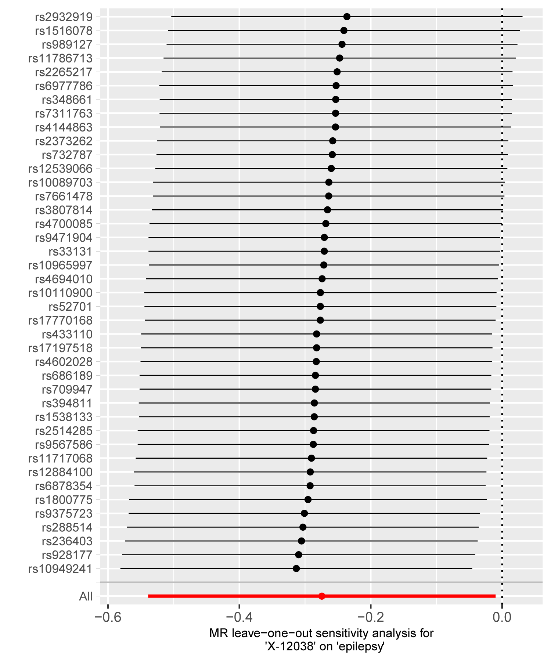

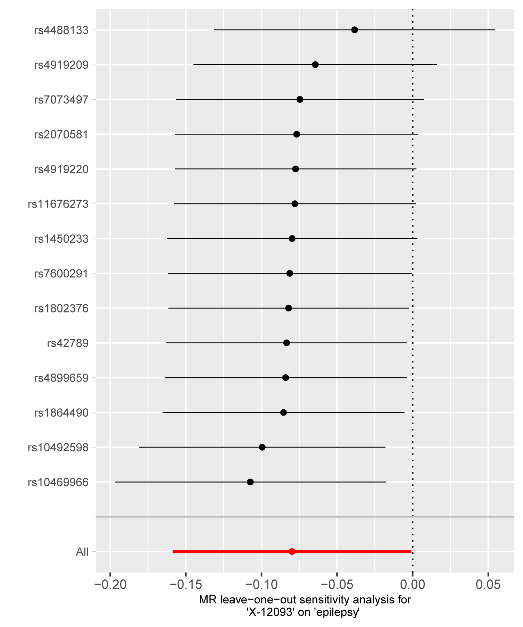

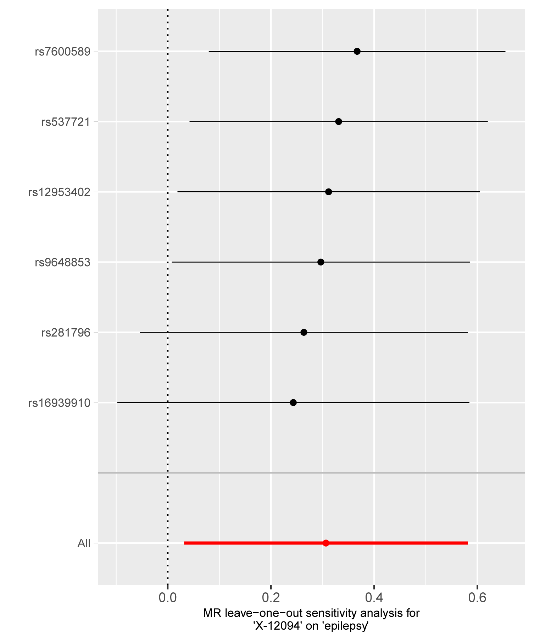

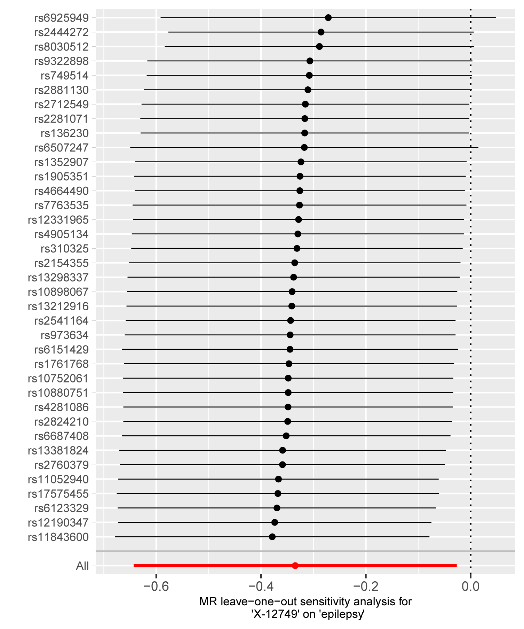

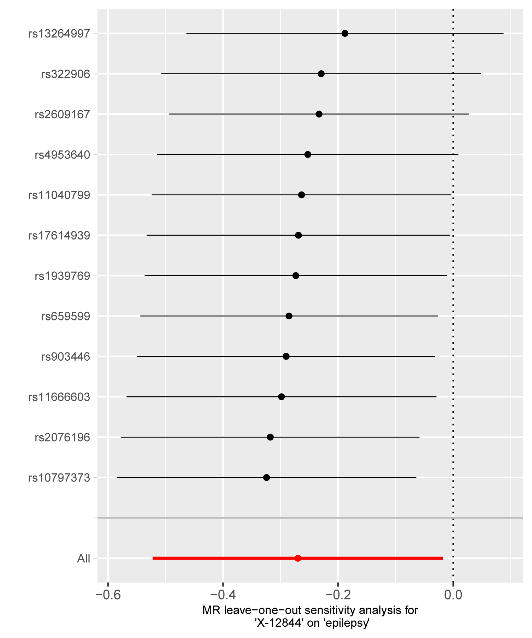

Supplement: Supplementary file 1 — Additional file 1: Figure S1. Forest plots for the Mendelian randomization (MR) leave-one-out analysis of the significant inverse variance weighted (IVW) estimates. Within each panel, the black points represent the causal estimate of the association between a specific metabolite and epilepsy after discarding each SNP in turn. Red points represent the pooled IVW estimates. Horizontal lines denote 95% confidence intervals. [file 12967_2022_3648_MOESM1_ESM.docx]
